# Black rain in Hiroshima: a critique to the Life Span Study of A-bomb survivors, basis of the linear no-threshold model

**DOI:** 10.1186/s41021-019-0141-8

**Published:** 2020-01-01

**Authors:** Shizuyo Sutou

**Affiliations:** School of Pharmacy, Shujitsu University, 1611 Nishigawara, Naka-Ku, Okayama-City, Okayama, 703-8516 Japan

**Keywords:** Fallout, Hermetic, Induced radiation, Linear no-threshold model, LNT, NIC, Not-in-the-city, Radiation hormesis, Residual radiation

## Abstract

Ionizing radiation is regulated by the linear no-threshold model (LNT), which asserts that the lowest doses of ionizing radiation are hazardous in proportion to the dose and dose rate. LNT is based on the data of the Life Span Study (LSS) of A-bomb survivors in Hiroshima and Nagasaki. Radiation doses of the survivors were estimated by using initial radiation (5% of blast energy) and residual radiation (10%) was neglected. The major component of residual radiation was fallout, most of which must be brought down to the ground by black rain. The rain was highly radioactive. There are three major black rain maps reporting that black rain covered wide areas of Hiroshima-City. The three lead to an important conclusion that not only A-bomb survivors but also not-in-the-city control subjects (NIC) were irradiated with residual radiation to a greater or lesser degree. This means that exposure doses in LSS were largely underestimated and that use of NIC as the negative control is faulty. Thus, LNT based on LSS is invalid. In addition, LSS ignores radiation hormesis ─ ionizing radiation is not always hazardous, but beneficial depending on doses and dose rates. Indeed, when LSS data of longevity were examined, a clear J-shaped dose-response, a hallmark of radiation hormesis, is apparent. Also, cancer mortality ratios are in the increasing order: NIC (exposed to residual radiation), A-bomb survivors (exposed to both initial and residual radiations), and the Japanese in general (no exposure). Thus, low dose radiation (LDR) is hormetic. Obstinate application of invalid LNT to regulation-unnecessary LDR has been causing tremendous human, social, and economic losses in Fukushima. Also, LNT prevents clinical application of radiation hormesis to age-associated diseases such as Alzheimer’s disease and cancers.

## Background

The tolerated dose of radiation exposure for workers had remained at 500 mGy/year from 1934 until 1956 with no difficulties, but the linear no-threshold model (LNT) was then recommended without solid supporting data by the National Academy of Sciences of the United States of America (NAS) [1]. Actually, LNT relies upon the assumption that even the lowest doses of ionizing radiation are hazardous in proportion to the dose. In 2006, NAS reported its support of LNT based on the Life Span Study (LSS) of Hiroshima and Nagasaki A-bomb survivors [2]. That report is invalid: 1) dose estimation was based only on the initially sustained doses (5% of total A-bomb energy), neglecting residual radiation (10%) and underestimating radiation doses; 2) control subjects (not-in-the-city control subjects, NIC, who entered Hiroshima and Nagasaki after A-bomb detonation) were assumed for the study to have zero exposure and zero risk of cancer, but they had been irradiated to a greater or lesser degree by residual radiation, compromising their status as negative controls; and 3) analyses were premised on the invalid LNT to emphasize hazardous effects of ionizing radiation, neglecting radiation hormesis (high doses are hazardous, but low doses are beneficial).

The three issues presented above are closely associated with black rain because black rain was the carrier of fallout, which was the major component of residual radiation. Black-rain-affected areas were so wide that most A-bombed people and NIC must have been irradiated with residual radiation. Some NIC died of acute radiation symptoms (ARS). Therefore, elucidation of black rain is indispensable to elucidate the effects on health from ionizing radiation from A-bombs. In 1957, Obo indicated clearly that the area around the epicenter in Hiroshima was heavily contaminated with residual radiation [3]. The reports appeared in a rather minor Japanese journal that the author had translated and summarized into English [4]. The Oak Ridge National Laboratory (ORNL) holds a document clearly illustrating that black rain readily induced ARS such as fever, vomiting, diarrhea, sore throat, and epilation [5]. However, the Radiation Effects Research Foundation (RERF), a Japan–US scientific organization and the center of LSS, is apparently reluctant to admit the effects of black rain [6]. Although the black rain issue involves many complicated factors such as time of onset, duration of rain, affected areas, intensity of rain, and intensity of radioactivity, intensive efforts have been made to clarify the nature of black rain. Its exact reproduction is difficult, however, mainly because data of scientific measurements were scant. Eyewitness accounts by A-bomb survivors constitute the major information sources.

Three major questionnaire studies are often cited in the context of black rain [7–9]. These three are mainly reviewed. Some other relevant data to support that black rain was radioactive and ARS-inducing and that LDR is hormetic in longevity and cancer induction in LSS are presented.

### Three major black rain maps

#### Uda map

**Fig. 1:**
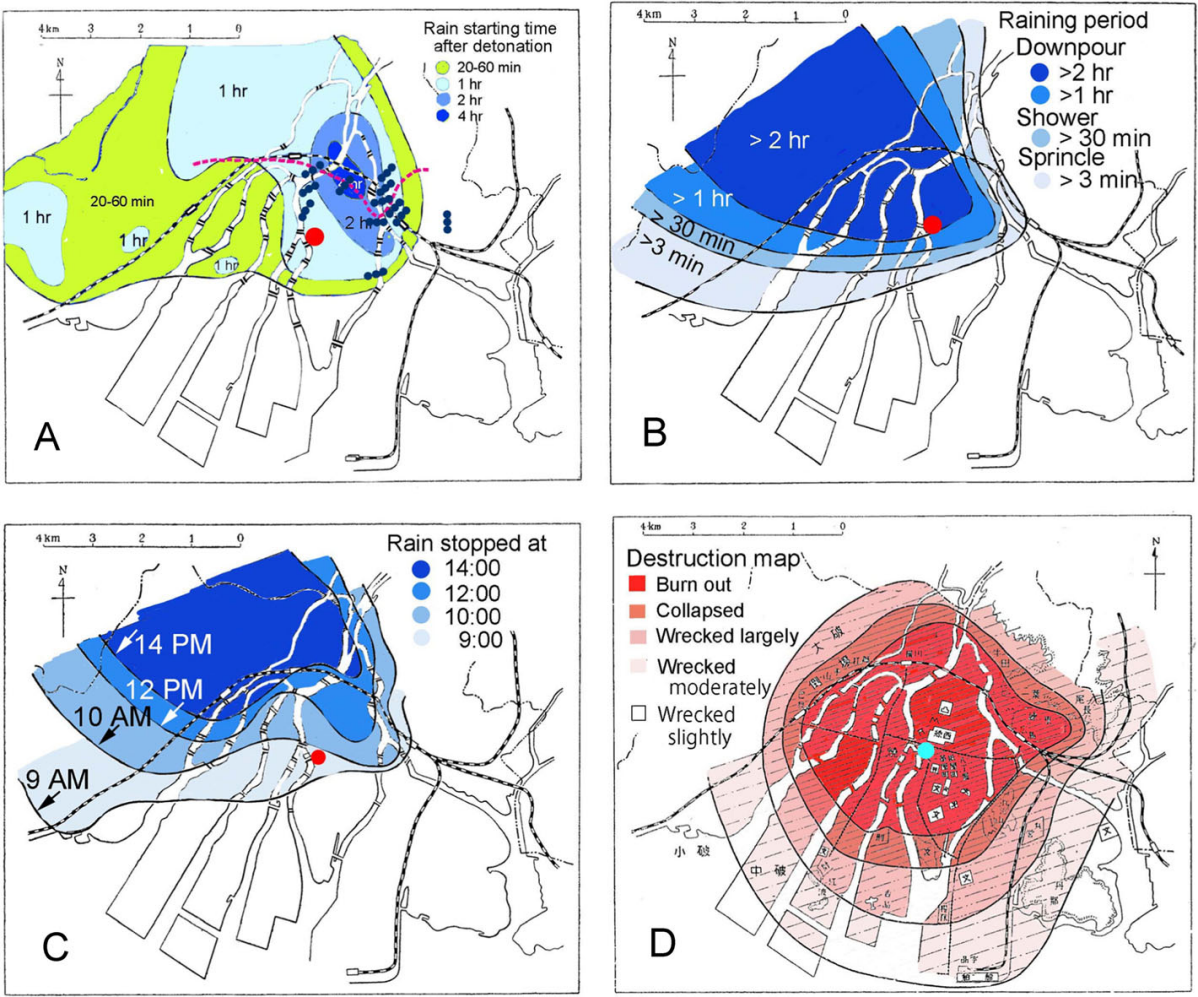
Uda map: **a**, rain starting time after detonation, wind front (pink dashed line), and whirlwinds (triple dots); **b**, rain period; **c**, rain stopping time; and **d**, destruction map. The epicenter is shown as a red circle in **a**–**c** and as a light blue circle in **d**. The original maps are colorless, but are colored above for ease of understanding

The Uda map is important for Hiroshima residents to obtain an A-Bomb Survivor Certificate from the Ministry of Health, Labour and Welfare (MHLW). Uda and his colleagues had worked for the Hiroshima Regional Headquarters of the Japan Meteorological Agency. They collected materials related to general conditions at the detonation, states of clouds and winds, whirlwinds induced by the ensuing inferno, rain showers caused by the detonation and inferno, thunder, falling objects, destruction of houses and buildings, and conditions of fires and burning [7]. The research was conducted during August–December, 1952, but Uda himself started data collection immediately after the bombing in 1945. Of a survey of at least 170 inhabitants, 116 testimonies are attached to the end of their article, from which four maps associated with black rain and destruction have come to be cited here (Fig. 1).

The Hiroshima A-bomb was detonated at 8:15 AM 600 m above the ground in the clear sky. Regarding the epicenter, black rain started at around 9 AM (Fig. 1A) and poured down torrentially for more than two hours (Fig. 1B). Figure 1C shows, however, that the rain stopped before 10 AM, meaning that the black rain lasted for about 1 h around the epicenter. The reason for this discrepancy is not known. Because Fig. 1B shows the blast center as located at the border of 1 h and 2 h areas, the actual raining period might be close to 1 h.

**Fig. 2:**
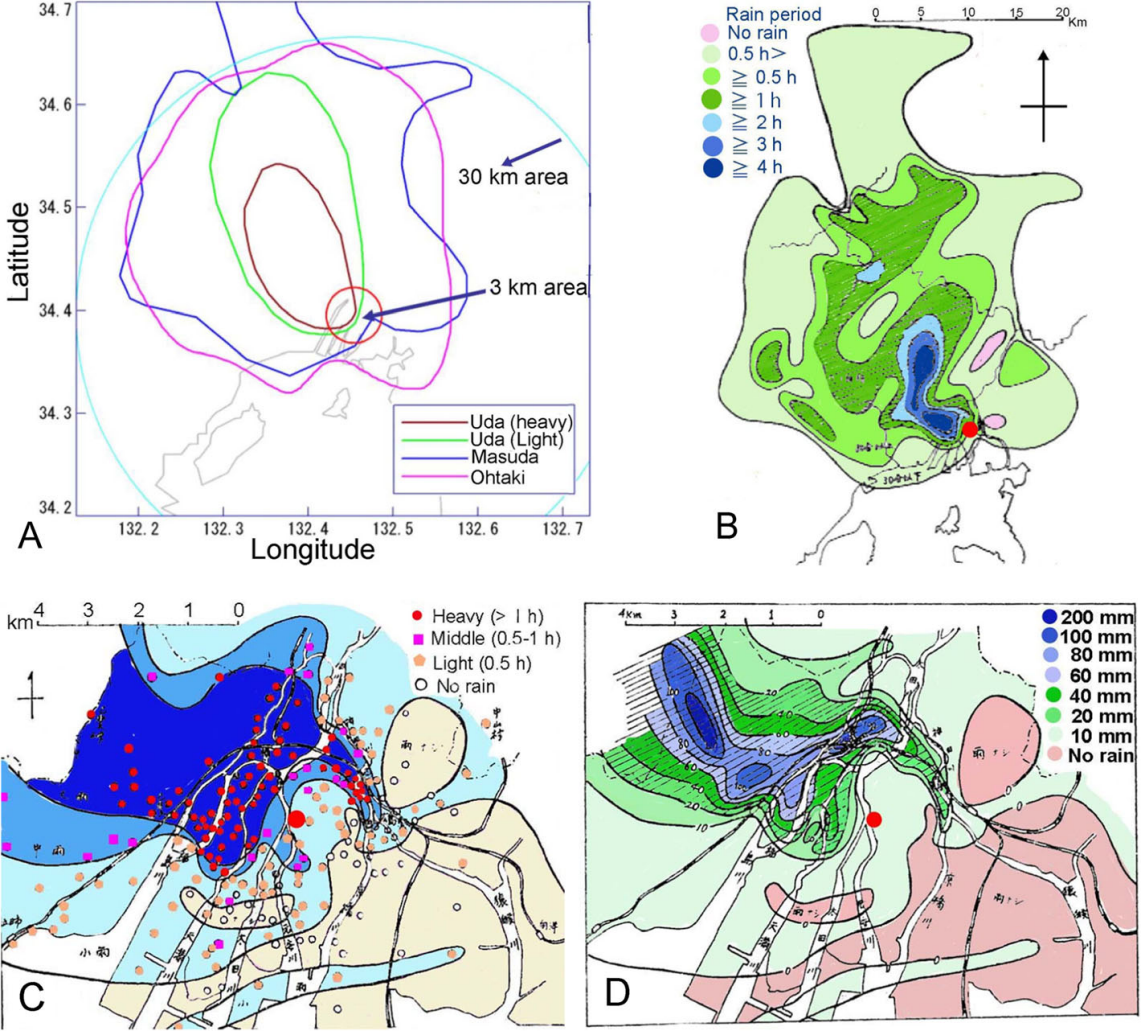
Rainfall maps: **a**, outlines of Uda, Masuda, and Ohtaki map; **b**, raining period in a large sale; **c**, rainfall in the central area of Hiroshima-City; and **d**, estimated volumes of rain in the central area. Degrees of rain were determined by the rain duration as follows: heavy, ≥1 h; middle, < 1 h – ≥30 min; and light, < 30 min (**c**). The original maps are colorless, but are colored here for ease of understanding

The rain started to pour around the epicenter and moved northwest. Ascending air currents produced by the A-bomb blast itself and resultant fire seemed to reinforce the rain synergistically. The heavy rain poured down in an oval area with the major axis of 19 km and minor axis of 11 km. The whole rain area including slight rain constituted an oval with the major axis of 29 km and minor axis of 15 km (Fig. 2A).

Initially, the rain contained black and sticky components. A rumor circulated that the black fluid must be oil spilt to burn Hiroshima-City, but it had neither the characteristics of oil nor an oily smell. Rushing black streams in the rivers produced foams. The naked skin hurt when huge rain drops as large as hail hit it. During the heavy rain, the temperature dropped drastically; people shivered with cold. Later analyses showed that the rain was radioactive. Fish such as carps in ponds and eels in rivers floated to the surface. Cattle had diarrhea. People in some areas had diarrhea for more than three months, probably because water pipes were destroyed. They drank well water or underground water. Harmful insects disappeared from rice fields. Rice plants showed abnormal growth, as if they had been treated with special fertilizers. Farmers looked forward to rich harvests. These latter observations hint at hormetic effects on plants. Typhoons struck this area twice on September 17 and 18 and on October 9 and 10, dooming their expectations of good harvests.

In heavy rain areas, black rain lasted for 1–2 h; then ordinary colorless rain came down. Components of the black rain seemed to contain the A-bomb fallout, with soil and debris produced by a Mach wave, and soot from fires. Black rain-affected areas were expected to be highly radioactive.

Actual rainfall was not measured, but heavy rain areas were flooded to an almost equal degree as that experienced from the typhoon on September 17 and 18, which suggests that the rain was 60–110 mm in 1–3 h. The total rainfall was estimated as 10–20 million m^3^. The rain was so heavy that some bushfires were extinguished by the rain alone.

During or after the rain, thunder rumbled several times around 10–11 AM. The thunder was peculiar, sounding like explosions or the roar of cannons. Thunder occurred in remote areas more than 20 km distance from the epicenter.

Black dust fell several kilometers outside of the rain areas, blackening the leaves of vegetables. Falling objects were galvanized iron sheets, wooden plates from roofs, pieces of mosquito netting, cotton rags, remnants of clothes, paper scraps, name cards, paper money, debentures, and so on. Of those, paper scraps including slips from governmental offices, banks, and post offices and strips of papers of account books were the most abundant. The areas of falling objects were distinct from the rain areas. The areas were southwest of the major axis from the epicenter and extended 30 km. Falling objects were carried at a speed of 1–3 m/s.

Hiroshima-City was destroyed concentrically from the epicenter to the outskirts (Fig. 1D). Fires started about 5 min after detonation in shattered houses and buildings that had been using open flames at the time of the blast. Others caught fire from the heat of the A-bomb blast. People were unable to the extinguish fires. The cumulative fires began to rage around 9 AM, becoming most furious from 10 AM to 2 PM, but abated in the evening. Most areas of Hiroshima-City were wrapped in smoke on the afternoon of August 6, 1945. The fire lasted for three days. Smoldering lasted for a weak. The heat of the A-bomb and inferno evaporated water from the rich waterways. Burning of organic materials produced water and CO_2_. Rain poured down from 9 AM to 4 PM.

Spontaneous fires were visible on straw roofs, straw in the fields, shrines thatched with *hinoki* bark, *shouji* screens, crude rubber, cement bags, the tops of electric poles, railroad ties, stakes beside railways, hemp, palms, and so on. Black objects collected heat more than white ones. A man dressed in white was burned slightly, but his wife in black beside him died from severe burns. People close to the epicenter were charred or evaporated. The skin of people between 0.5–2 km sometimes peeled off (drawings can be viewed at the Hiroshima Peace Memorial Museum (HPMM)). Most people outdoors at 2–4 km from the center were burned slightly, as though they had been suntanned.

Most people outdoors at 1–2 km felt violent heat. Those at 4–5 km felt hot. Some people 20 km away from the epicenter felt warm. Leaves of plants such as rice plants, corns, eggplants, pumpkins, lotuses, and sweet potatoes and trees such as pine, oak, cherry, and holly were charred or burned depending on their distance from the epicenter. Many trees died of heat at 2.5 km from the epicenter. Trees were partially burned at 4 km from the epicenter. It was midsummer, but colored leaves of wooded hills impressed people as if it were fall. After one month or so, new shoots emerged. Then the normal landscape was recovered.

The time of detonation was close to a morning calm: weakening land winds were blowing from the north in the northern parts of Hiroshima-City, with growing sea winds in the southern parts from the central area of the city, forming a front close to the railway (Fig. 1A). The epicenter was located south of the front. Strong sea winds blew into fierce ascending air currents after the detonation and big fire. Great whirlwinds (Fig. 1A) appeared along the front between 11 AM and 15 PM. Some whirlwinds developed into tornados. Drums, thick wood boards, galvanized iron sheets, suitcases, beer bottles, water, some people, and other objects were whirled upward.

Uda’s house was located in Takasu Area, a heavily contaminated area. Wooden sliding shutters were blown out into the garden and were pelted by black rain. When radioactivity of a muddy sample was measured at RIKEN, it was 50 times higher than the natural background level even 2 months after the blast. When his second son came back home from the evacuated place 3 months after the blast and slept beside the contaminated sliding shutters, his hair began to fall out. Astonished, Uda soon removed the shutters.

#### Masuda map

A new law, “Act for Atomic Bomb Sufferers’ Medical Care,” was passed in 1957 to support the A-bomb survivors medically. The survivors were issued an A-Bomb Survivor Certificate by the Ministry of Health, Labour and Welfare (MHLW). This law had been revised several times. In 1965, parts of “Heavily Contaminated Areas with Residual Radiation” were designated as “Specially Irradiated Areas.” This change opened a path to supporting people living in black rain-affected areas. The Specially Irradiated Areas, however, were so limited. In 1968, another new law, “Act on Special Measures of the Atomic Bomb Survivors,” was enacted to assist survivors’ livelihood in addition to medical measures. People asked Hiroshima-City and Hiroshima-Prefecture to expand the Specially Irradiated Areas. In 1976, the heavy rain area of the Uda map (Fig. 2A) was designated as “Special Areas for Physical Examination.” When specified illnesses were detected, the relevant person was regarded as an A-bomb survivor and was given an A-Bomb Survivor Certificate. In 1978, “the Association of Liaison Committees of Hiroshima-Prefecture of ‘Black Rain and Home Nursing’ A-bomb Victims” (Black Rain Association) was established. This group pushed for expansion of the Special Areas for Physical Examination.

Masuda used to work for the Meteorological Research Institute, the Japan Meteorological Agency. He learned through the Black Rain Association that the Uda map was not always correct, e.g., black rain dropped outside the rain areas that Uda and his colleagues had determined. He decided to reconstruct the black rain areas using various materials. He collected data for three years from 1985. 1) Weather data of August 6, 1945, were collected by the Hiroshima Regional Headquarters such as temperature, wind velocity, wind direction, rainfall, and cloud cover. 2) Uda and his colleagues recorded 116 testimonies [7], but they required more materials. Masuda was able to find and use a total of 170 materials that Uda et al. had used and kept at Tokyo University of Marine Science and Technology. 3) In 1974, Hiroshima-City and Hiroshim-Prefecture conducted a questionnaire survey of black rain. From the ca. 20,000 subjects, 17,369 responses were collected, but most were lost; 1045 were available. 4) Just before the establishment of the Black Rain Association, its later Chairman Hanamoto administered a questionnaire to 243 families, of whom 83 responded. 5) In 1978 and 1979, Secretary-general Murakami of the Black Rain Association convened meetings at 17 places and interviewed residents about black rain. He left a map on a scale of 1:50,000 in which black rain information of 25 areas was available. 6) On June 13 and 14, 1987, Masuda, in cooperation with the association, had meetings at five locations where a total of 340 people gathered, 72 of whom provided him with relevant information. On August 5, 1987, interviews were conducted at 10 places, yielding 111 testimonies. 7) In cooperation with the Association, a questionnaire survey was administered; 1188 answers were collected. 8) Newspaper articles and interviews on TV were collected; a total of 61 data were adopted. 9) Memoirs and recordings describing experiences were collected from 37 books and journals; 358 data were obtained.

Masuda collected many data to produce the Masuda map (Fig. 2A) [8]. The period during which data of papers were accepted was 1945–1988. During about 43 years, memories might become dim. In the early days after the A-bombing, people tended to conceal the fact that they were A-bomb survivors because they were sometimes discriminated against socially, e.g., engagements were often canceled. When the heavy rain area of the Uda map (Fig. 2A) made in 1953 was designated as “Special Areas for Physical Examination” in 1976, some exaggerated responses were expected thereafter, given so that a person could obtain a certificate.

When the slightest rain was included, the black rain area was 1250 km^2^ with 45 km north–northwest length and 36 km east–west width, ca. four times larger than the Uda map area (Fig. 2A, B). Rain areas are more complicated than those of the Uda map. A marked difference between the two maps is that the epicenter is in the heavy area in the Uda map (Fig. 1B), but is in the light area in the Masuda map (Fig. 2C). Because the rain started after 1 h from the detonation (Fig. 1A) and stopped before 10 AM (Fig. 1C), the rain must have lasted for less than 1 h. Therefore, the Masuda map (Fig. 2B) must be correct. Uda might think that the rain must be heavy in the central area. Figure 2D shows the deduced rain volumes. The northwest parts to the epicenter received heavy rains.

The Masuda map was examined by an expert committee established by Hiroshima-City and Hiroshima-Prefecture. It consisted of 12 members, but Masuda was excluded. In May, 1991, after 10 meetings over two years, the conference concluded that the Masuda map could not be adopted as the black rain map because the effects of black rain on humans had not been established. His map is not shown in HPMM.

#### Ohtaki map

In 2008, Hiroshima-City conducted a questionnaire survey of about 37,000 residents who might have experienced black rain. The rain start time, stopping time, and location were asked and 23,780 people responded. Effective responses were limited: those with both start and end times were 1084; those with only a start time were 481. Analyses indicated that black rain started about 9 AM, reached its heaviest level of precipitation at around 10 AM, spread northwestward from the epicenter, and disappeared around 30 km north–northwest from the hypocenter at 3 PM. The black rain area was estimated as 5–6 times greater than the Uda map and slightly greater than the Masuda map (Fig. 2A) [9].

The Ohtaki map entails some issues. Data were obtained from a survey sponsored by Hiroshima-City. The mayor must plan to support citizens sincerely to the greatest extent possible, but a politician must think carefully about voting behavior. The survey was administered 53 years after the A-bombing; responders’ memories might be vague. Many or most respondents, the vast majority of whom were 60 years old and older, must have wanted to have a certificate allowing them certain health and other benefits. A tendency to exaggeration of responses can be inferred.

The Ohtaki map was examined by the MLHW, which concluded that specification of black-rain-areas was difficult. This map has been displayed in HPMM since 2010 (see Fig. 2 (right) in an earlier report by the author [10]).

### Black rain was radioactive

#### Proceedings of new radiation dosimetry system DS02 of the atomic bombing

Proceedings of the workshop ‘new radiation dosimetry system DS02 of the atomic bombing in Hiroshima and Nagasaki’ include several reports describing that black rain was radioactive [11]. Shizuma measured three samples: 1) 28 soil samples collected by Nishina of RIKEN within 5 km from the epicenter on August 9, 1945; 2) 117 rock samples stored at the Petrological Laboratory, the Department of Science, Hiroshima University; and 3) black rain streaks on wall panels [12]. He was able to detect the γ-ray spectrum of Cs-137 in all three samples, indicating that fallout carried down by black rain contained an A-bomb product.

Yashima’s house was located in the Takasu area, 3.2 km west to the epicenter. The roof of his house was shifted by the blast wind, leaving a gap between the roof and the room. Black rain came into the room, leaving many black streaks on the wall panels. The rain was sticky. The streaks showed slight thickness. Two wall panels were given to HPMM, where they can be viewed. U-235 was used to make the Hiroshima A-bomb. The ratio of U-235/U-238 was expected to be higher than 0.00736, the natural ratio. Indeed, the rain samples showed slightly higher ratios than 0.00736 [13].

#### Obo report indicates hazardous effects by residual radiation

Obo had been running his hospital in Hiroshima-City. He wanted to ascertain the relation between the A-bomb radiation and cancer. A-bomb survivors (3946) and NIC (629) who lived in the area 0–7.0 km from the epicenter were interviewed in January–July 1957 with the help of Hiroshima University students. Examinees were asked about the place at the A-bombing, presence or absence of ARS, and if they entered the central area with a radius of 1 km from the epicenter within 3 months after the blast. The survivors were divided into two groups: those bombed outdoors and indoors. Each group was divided further into two subgroups: those who entered the central area (entrants) and those who did not (non-entrants). NIC were divided into entrants and non-entrants [3, 4].

Radiation from the A-bomb can reach 3 km from the epicenter, but 50–60% outdoor and 20–40% indoor bombed survivors showed ARS, indicating hazardous effects of residual radiation. Non-entrant survivors residing beyond 4.5 km showed a few percent of ARS irrespective of the outdoor or indoor bombing, but about 30% entrant survivors experienced ARS without regard to outdoor or indoor bombing, indicating strong residual radiation in the central area. More direct evidence of residual radiation was found: around 10–50% NIC entrants showed ARS within three weeks, depending on the entrance date. No ARS, however, were visible in NIC non-entrants. Among the areas of residual radiation, the induced radioactivity was limited to a 2 km radius from the epicenter. It disappeared in a few days. Therefore, the hazardous effects outside the 2 km circle must be attributable to fallout included within the black rain.

#### Committee for Approval of survivors estimates residual radiation

The Committee for Approval of Survivors presented the criticism that exposure doses of survivors estimated from the initial radiation only were greatly underestimated beyond 1.5 km [14]. In fact, the ARS of residents in remote areas and NIC cannot be explained without the effects of residual radiation. The Committee examined and analyzed the Obo report [3] and estimated the average exposure doses of NCI: 1.5 Gy (equivalent to the initial radiation at 1200 m from the epicenter) on the A-bombing day; 0.7 Gy after a week; 0.35 Gy after two weeks; 0.18 Gy after three weeks; and 0.09 Gy after one month.

#### Technical report of oak Ridge National Laboratory: ORNL-TM-4017

The census taken in 1950 revealed that there were about 280,000 A-bomb survivors in Japan and that around 200,000 lived in Hiroshima or Nagasaki. The master sample questionnaire (MSQ) was administered to 200,000 people during 1956–1961. The results became basic data, from which about 93,000 were chosen for LSS. MSQ was applied to 27,000 NIC. Other MAQ data were added to reach totals of 138,000 and 23,000, respectively, in Hiroshima and in Nagasaki.

During 1954–1965, 20,356 Hiroshima and 8373 Nagasaki survivors were interviewed for their shielding histories. Rain (when, where, what kind, etc.) was included in question items until a format change in September, 1958. Results were recorded on microfilm. The copies were stored in ORNL, which held 11,915 Hiroshima and 2046 Nagasaki survivors’ records. These survivors were more than 1600 m from the epicenter. Yamada and Jones selected 222 black rain-exposed Hiroshima survivors from the microfilm data. Their radiation symptoms were extracted from MSQ data. Separately, a database was constructed by combining the MSQ data and shielding histories. They were called the ABCC magnetic tape records. This contained 75,100 Hiroshima and 24,900 Nagasaki survivors, from which black-rain-exposed 65 Hiroshima subjects and 16,045 control people who lived in southeast portion of Hiroshima were selected. Consequently, a total of 287 survivors were extracted to ascertain the effects of black rain. A technical report was produced: ORLN-TM-4017 [5]. The original is kept at the Department of Energy.

Major findings extracted from the report are shown in Table 1. Even though the rain-exposed population was small (287), the hazardous effects of black rain are clear. The authors seemed to urge implicitly RFRF as relevant data to reflect the black rain effects.

**Table 1:** Specific radiation symptoms versus initial exposure

Symptoms with onset within 21 days	Black rain survivors^a^	Control survivors^b^
Fever	32 (13.56%)	212 (1.32%)
Vomiting	18 (7.62%)	88 (0.55%)
Diarrhea (non-bloody)	39 (16.53%)	122 (0.76%)
Diarrhea (bloody)	13 (5.51%)	37 (0.23%)
Oropharyngeal lesions		
Sore throat	13 (5.51%)	212 (0.13%)
Sore mouth	10 (4.24%)	14 (0.09%)
Sore gums	5 (2.12%)	7 (0.04%)
Purpura		
Gingival bleeding	11 (4.66%)	25 (0.16%)
Purpura	7 (2.97%)	14 (0.09%)
Other bleeding	1 (0.43%)	1 (001%)
Non-purpuric bleeding	11 (4.66%)	231 (1.44%)
Epilation		
Slight (less than 1/4)	142 (60.17%)^c^	646 (4.03%)
Moderate (1/4 to 3/4)	13 (5.51%)	47 (0.29%)
Sever (more than 3/4)	2 (0.85%)	20 (0.12%)
Present, but not specified	5 (2.11%)	12 (0.07%)

^a^, Initial exposure: < 10 mGy, 74; 10–100 mGy, 121; 100–200 mGy, 41; and > 200 mGy, 51

^b^, Initial exposure: < 200 mGy

^c^, Some cases without epilation were apparently added erroneously to the slight epilation group and the actual number must be less than 142 [15]

#### Induced radiation

The A-bomb blast released a huge amount of energy: thermal radiation (35%), blast energy (50%), and nuclear radiation (15%). Of the 15, 5% was the initial radiation released within 30 s. It was used to estimate the exposure doses. The remaining 10% was residual radiation, which consisted of induced radiation and fallout. Fallout accounted for most of the residual radiation. Hazardous effects of fallout on survivors and NIC might be readily apparent, but quantitative estimation has been quite difficult mainly because of the complicated rainfall status and the lack of reliable radiation data.

Induced radiation is produced by the action of neutrons in making non-radioactive substances into radioactive ones. The extent of radiation from the A-bomb was ca. 3 km from the epicenter, but the extent of neutrons was limited to 2 km. Induced radiation can be determined more precisely than that of fallout. Half-lives of induced nuclides were short and fell off rapidly with distance from the epicenter and with time. The maximum cumulative dose was estimated as 800 mGy [16]. The dose would be about one-third as large after a day and only a few percent after a week. For example, a person entering the Hiroshima epicenter area after 1 day and working 10–20 h a day for a week would have been exposed to about 100 mGy. If the person had been working at a distance of 500 m, then exposure would have been 10 mGy. On August 6, 1945, 84 NIC entered the central area and 40 people (47.6%) showed ARS [3, 4]. When people stayed in this area for 1 h, 4 h, 1 day, 2 days, and 4 days, the respective percentages with ARS were 11.7, 28.6, 42.5, 50.0, and 70.0. These people were definitely irradiated by both induced and fallout radiation.

### A-bomb survivors get Cancer less frequently and live longer

#### The reason why BEIR VII report is flawed

NAS put forward the BEIR VII report based on a LSS study in 2006 as evidence supporting LNT. This report seems to have been intended from the start to support the old LNT, ignoring a half century of scientific development. Much evidence indicates that black rain carried down fallout onto wide areas and that it caused hazardous health effects on inhabitants outside the reach of the initial radiation. Therefore, exposure doses are underestimated. Main flaws in the BEIR VII report include the following:

- The dose-response of leukemia is clearly linear-quadratic. That of solid cancers fits better to a linear-quadratic than linearity, but no statistical significance was found between the two; the BEIR VII report adopted linearity. This forced logic is unacceptable.
- The highest dose in Fig. 4 of the BEIR VII report is 2 Gy. Excess relative risk (ERR) usually shows a downturn of more than 2 Gy because highly exposed people would die before cancer development. Concealing a downturn by limiting doses up to 2 Gy, the BEIR VII report is successful in giving the impression of linearity.
- The BEIR VII report combined all data < 100 mGy into one data point. More than 80% of all survivors belong to this < 100 mGy category. Combining data into arbitrary groups is often said to be a statistical trick. Such combination is successful in giving the impression of linearity.
- The BEIR VII report is based on the false assumption that zero dose exposure and zero ERR in NIC, but this is invalid because most NIC were exposed to residual radiation. Setting aside this assumption, Bayesian analysis of LSS shows a threshold and hormetic response [17].
- Living organisms, including humans, have defense mechanisms against ionizing radiation such as reactive oxygen species (ROS) quenching, DNA repair, apoptosis, and anticancer immunity. The BEIR VII report ignores these mechanisms acquired through the evolutional processes of billions of years. The report was based on faulty assumptions [18].

#### A-bomb survivors get cancer less frequently

RERF publishes an LSS report once every five years. The quantities of A-bomb survivors and solid cancer deaths were extracted from the latest three articles and were compared with Japanese national averages (Table 2). Rates of cancer deaths in both A-bomb survivors and NIC are lower than those of Japanese nationwide. The finding is readily acceptable when one acknowledges that low-dose radiation is hormetic under appropriate conditions and that both A-bomb survivors and NIC were exposed to hormetic radiation. Approximately 95% survivors had been exposed to < 0.5 Gy (probably in the hormetic range).

**Table 2:** Solid cancer mortality in the lifespan study of A-bomb survivors compared to Japanese cancer mortality. Japanese average cancer deaths were calculated by dividing cancer deaths by total deaths each year during 1958–2009 [19]

Reporters	Year	Survey period	No. *hibakusha* or [NIC]	No. cancer deaths (%)	% Japanese average cancer deaths
Preston et al. [20]	2007	1958–1998	105,427 [25,427]	17,448 (16.6^b^) [3994 (15.7^b^)]	21.4 (1958–1998)
Ozasa et al. [21]	2012	1958–2003	86,611 [26,529]	10,929 (12.6^b^) [NA^a^]	22.3 (1958–2003)
Grant et al. [22]	2017	1958–2009	80,205 [25,239]	17,316 (21.5^b^) [5222 (20.6^b^)]	23.3 (1958–2009)

^a^, not available; ^b^, statistically significant at *P*<0.01 (chi-square test)

#### A-bomb survivors live longer

**Fig. 3:**
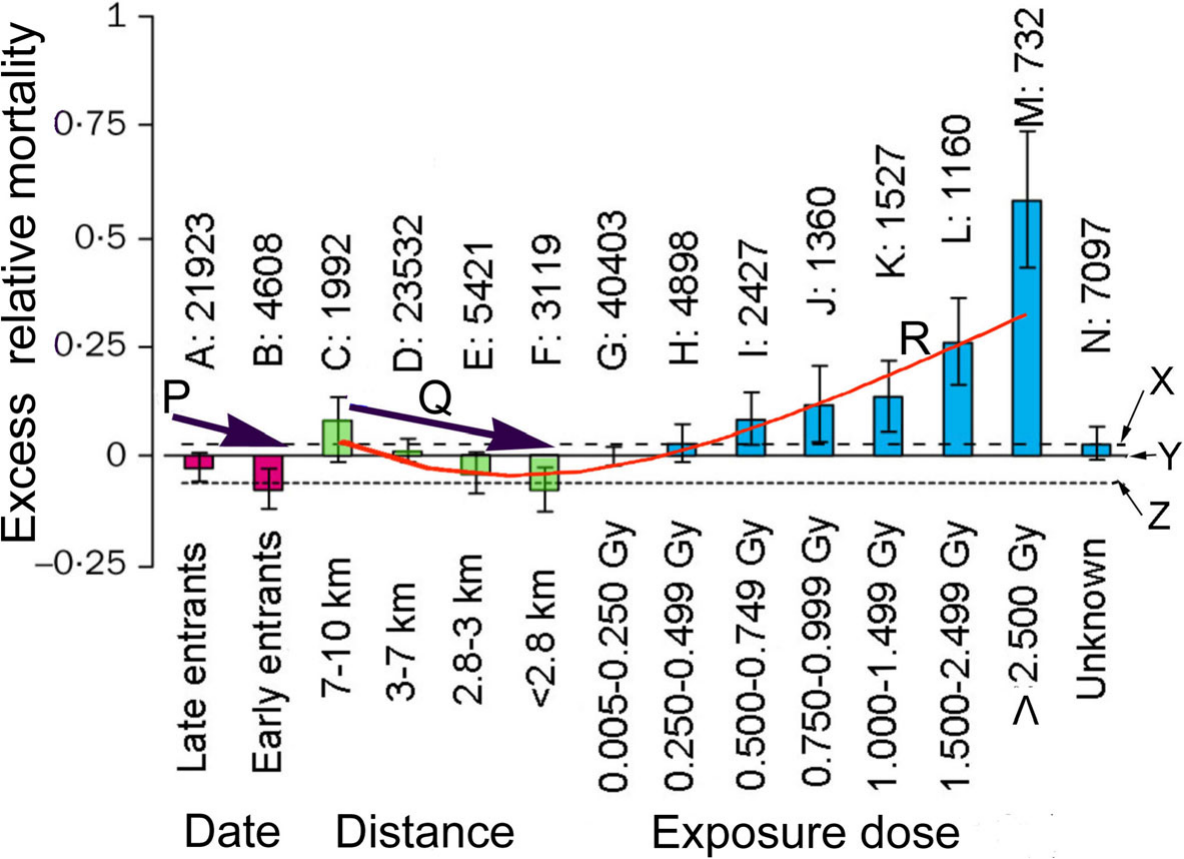
Excess relative mortality by entrance date, distance from the hypocenter, or radiation dose. Figure 1 and Table 1 of an earlier report [24] are combined. A, late entrants (NIC, entered after 1 month); B, early entrants (NIC, entered within 1 month); C–F, < 0.005 Gy dose groups with different distance from the epicenter; G–M, different dose groups; and N, people (0.11–3 km) with unknown doses. Numerals shown above A–N denote the number of people studied. Solid lines X, Y, and Z respectively represent the averages of C and D (*n* = 25,524), of C–F (*n* = 34,064), and of E and F (*n* = 8540)

Stewart and Kneale [23] showed that deaths in 1950–1982 from all non-malignant diseases in LSS population were significantly lower in survivors exposed to low doses than in unexposed persons. Cologne and Preston [24], however, concluded that the lifespan of A-bomb survivors was shortened. They assumed zero exposure and zero hazardous effects in NIC, the negative control cohort. Analyses were made based on LNT. Their data are reproduced in Fig. 3.

We can draw the conclusion that A-bomb survivors live longer, on average, from Fig. 3.

- Excess relative mortality of early entrants (Fig. 3A) is greater than that of late entrants (Fig. 3B), indicating that people with higher exposure lived longer than those with lower exposure (arrow P). The key to resolving this contradiction is the inference that radiation hormesis stimulates human bodily defense mechanisms.
- Excess relative mortality is inversely proportional to distance from the epicenter (arrow Q), again indicating that people with higher exposure lived longer than those with lower exposure.
- Excess relative mortality shows a typical J-shaped curve (arrow R), a hallmark of radiation hormesis. The crossing of the J and the x-axis implies a threshold at around 0.250–0.499 Gy (Fig. 3, H). When residual radiation is examined, the threshold is expected to be around 1 Gy. Survivors in Hiroshima were irradiated acutely, while people in Fukushima were don chronically; the threshold in Fukushima would be more than 16 times larger than that in Hiroshima [25].

### Water source of black rain

Reportedly, about 1 g of U-235 was converted to energy during the Hiroshima A-bomb explosion. According to the eq. *E* = *mc*^2^, 1 g of mass produces an energy of 9 × 10^13^ J/g. A radius of 2 km area (1.25 × 10^7^ m^2^) was burned down in Hiroshima-City (Fig. 1D). Maruyama and Yoshikawa deduced that houses made of wood had been built with a 30% coverage ratio (3.76 × 10^6^ m^2^) and that the unit of mass was 100 kg/m^2^, giving a mass of 3.76 × 10^11^ g [26]. We assume that the wood consisted of cellulose made from glucose. Because the heat of combustion for glucose is 1.55 × 10^4^ J/g, the heat produced by the burned houses is estimated as 5.82 × 10^15^ J, which is 65 times greater than that produced by the A-bomb. Therefore, the major destructive power of the Hiroshima disaster was not represented by the A-bomb itself, but by anthropogenic organic materials such as houses, buildings, and human bodies. The Hiroshima inferno was ignited by the A-bomb.

A lot of water was produced by the inferno. It contributed to the formation of black rain. The molecular weight of glucose is 180, so 3.76 × 10^11^ g means 2.08 × 10^9^ mol. Because 1 mol of glucose produces 6 mol of water, 1.25 × 10^10^ mol water was produced. The molecular weight of water is 18, 2.25 × 10^11^ g or 2.25 × 10^5^ t of water were produced. About 70% of a human body consists of water, so a person with 70 kg has 50 kg water. When the remaining parts of a body are burned, H_2_O, CO_2_, and other inorganic compounds are produced. There were many people, animals, trees, and other objects and organisms burned and charred, thereby producing water. The large amount of water thus produced must have contributed to make the black rain that carried most fallout down onto the ground.

Rainfall around the epicenter was apparently limited (Fig. 2C, D). This does not mean, however, that radioactivity levels around it were low because the initial rain might be fallout-rich. One can infer that NICs who experienced ARS (Fig. 3 in an earlier report by the author [4]) must have been irradiated heavily with residual radiation, whereas those who lived long lives (Fig. 3) or who were unaffected by cancer (Table 2) must have been irradiated with a hormetic range of radiation.

### Two dark sides of LNT

People in the restricted area with the radius of 20 km from the Fukushima Daiichi Nuclear Power Plant were forced to evacuate. The quake and tsunami victims of the Great East Japan Earthquake in Fukushima was 1607, while the number of disaster-associated deaths was 2272 as of March 31, 2019 [27]. Of 2272, 1793 were the Fukushima accident-associated deaths according to the Fukushima Minpo. Thus, the forced evacuation claimed more victims than the quake and tsunami. No one was killed by radiation itself. Therefore, it is obvious that too conservative, unnecessary restriction of LDR based on LNT has killed so many people.

LDR is hormetic. This is demonstrated by a lot of in vivo and in vitro experiments using animals, cultured cells, and other organisms. Table 2 and Fig. 3 show radiation hormesis in LSS [10]. Solid cancer deaths in Hiroshima A-bomb survivors [28] and leukemia deaths in LSS [29] are shown to be hormetic. Some clinical applications of radiation hormesis to non-Hodgkin lymphoma [30], Alzheimer’s disease [31], four solid cancers [32], and melanoma [33] have been reported, but clinical cases are quite limited. LNT is preventing full development of hormetic therapy for age-associated diseases such as Alzheimer’s disease, Parkinson’s disease, dementia, and cancers.

## Conclusion

LNT asserts that the lowest doses of ionizing radiation are hazardous in proportion to the dose and dose rate. LNT is supported by LSS, in which radiation doses were estimated by using initial radiation (5% of blast energy) and residual radiation (10%) was neglected. The major component of residual radiation was fallout, most of which was brought down to the ground by black rain. There are three major black rain maps reporting that black rain covered wide areas of Hiroshima-City. The rain was highly radioactive. Therefore, not only A-bomb survivors but NIC were irradiated with residual radiation. This means that exposure doses of A-bomb survivors were largely underestimated and that use of NIC as the negative control is faulty. Thus, LNT based on LSS is invalid. In addition, LSS ignores radiation hormesis ─ ionizing radiation is beneficial at lower doses. Indeed, A-bomb survivors live longer and get cancer less frequently. Thus, LDR is hormetic. Notwithstanding, LNT is the basis of radiation regulation now and causing tremendous human, social, and economic losses and preventing clinical application of radiation hormesis to age-associated diseases such as Alzheimer’s disease and cancers. It is high time to admit beneficial effects of radiation hormesis and to establish a new paradigm for LDR regulation.

## Data Availability

All data generated or analyzed during this study are included in this published article.

## Abbreviations

ARS: acute radiation symptoms
ERR: excess relative risk
HPMM: Hiroshima Peace Memoriam Museum
LDR: low dose radiation
LNT: linear no-threshold model
LSS: Life Span Study
MHLW: Ministry of Health, Labour and Welfare
MSQ: master sample questionnaire
NAS: National Academy of Sciences of the United States of America
NIC: not-in-the-city control subjects
ORNL: Oak Ridge National Laboratory
RERF: Radiation Effects Research Foundation
